# Comparison of Different Non-invasive Indices in Predicting High-Risk Esophageal Varices in a Pakistani Population

**DOI:** 10.7759/cureus.81507

**Published:** 2025-03-31

**Authors:** Abdul Wahid Balouch, Khaild Tareen, Ali Hyder, Imran Ahmed, Azhar Ali, Muhammad Usama Kayani, Raja Taha Yaseen Khan, Nasir Hassan Luck

**Affiliations:** 1 Department of Hepatogastroenterology, Sindh Institute of Urology and Transplantation, Karachi, PAK; 2 Department of Gastroenterology, Sheikh Khalifa Bin Zayed Al Nahyan Medical Complex, Quetta, PAK; 3 Department of Gastroenterology, Chandka Medical College, Shaheed Mohtarma Benazir Bhutto Medical University, Larkana, PAK; 4 Department of General Internal Medicine, King’s College Hospital, London, GBR; 5 Department of Gastroenterology, Sindh Institute of Urology and Transplantation, Karachi, PAK

**Keywords:** cirrhosis, evendo score, high-risk varices, non-invasive indices, portal hypertension

## Abstract

Introduction

Esophageal varices (EV) generally develop as a complication of portal hypertension, whereas high-risk esophageal varices (HRV) are associated with significant morbidity and mortality. Although endoscopic surveillance is widely recommended in recent guidelines, its invasiveness and cost are important concerns, particularly in resource-poor countries such as Pakistan. The aim of this study was to compare the diagnostic performance of the P^2^MS index ((platelet count (×10^9^/L))^2^ / (monocyte fraction (%) × segmented neutrophil fraction (%)), the EVendo score, the international normalized ratio (INR) to platelet ratio (INPR), and splenic stiffness for predicting HRV in a Pakistani population.

Study methodology

After the approval from the Ethical Review Committee, Sindh Institute of Urology and Transplantation (ERC-SIUT) (approval-1141), this cross-sectional study was carried out at the Department of Hepatogastroenterology, Sindh Institute of Urology and Transplantation, Karachi, from April to September 2024. A total of 340 cirrhotic patients aged >18 years underwent laboratory tests, abdominal ultrasound, shear-wave elastography (SWE), and esophagogastroduodenoscopy assessment of HRV. Data were analyzed for the area under the receiver operating curve (AUROC) for each non-invasive index in predicting HRV, and at an optimal cutoff, sensitivity, specificity, and diagnostic accuracy of these indices were obtained.

Results

Among 340 patients, HRV was detected in 84 (24.7%). The EVendo score showed the highest AUROC of 0.92 (p ≤ 0.001) with a diagnostic accuracy of 86.56%, followed by the P^2^MS index, which had an AUROC of 0.859 and diagnostic accuracy of 81.71%, splenic stiffness with an AUROC of 0.838 and diagnostic accuracy of 66.47%, and INPR with an AUROC of 0.90 and diagnostic accuracy of 58.24%. EVendo score was associated with higher sensitivity, 92.94%, and specificity, 71.88%, for predicting HRV.

Conclusion

Non-invasive indices, mainly the EVendo score, showed a very good diagnostic performance for predicting HRV in resource-limited settings like Pakistan. These tools might have the potential to restrict indications for routine endoscopy, thus improving patient management and reducing the cost and economic burden. Future multicenter studies will be required for the validation of these results and to enhance their clinical applicability.

## Introduction

Among the major concerns of hepatology, portal hypertension carries a myriad of serious complications, including esophageal varices (EV) [1]. Variceal bleeding is a dangerous consequence of EV and constitutes considerable morbidity and mortality [2]. Early identification and monitoring of high-risk esophageal varices (HRV) are important for the mitigation of these outcomes [3,4]. Current guidelines recommend periodic endoscopic surveillance for the detection of EV [5]. The invasiveness, added to the high cost and risks of endoscopy, nonetheless constitutes grounds for many patients to avoid the procedure, especially in resource-constrained settings like Pakistan [6].

Over the years, there has been a move toward non-invasive means of predicting HRV as an alternative to endoscopy. Several predictive models have been suggested, including the P^2^MS ((platelet count (×10^9^/L))^2^ / (monocyte fraction (%) × segmented neutrophil fraction (%))) index, EVendo score, Liaoning score, and splenic stiffness [6-9]. These indices use clinical, biochemical, and radiological parameters in an effort to stratify patients at risk with the aim of limiting endoscopy to high-risk cases only [8]. These non-invasive techniques, in particular, provide very encouraging alternatives to endoscopy, especially in resource-poor settings, and may aid in the early detection and management of HRV [10].

Chronic liver diseases due to hepatitis B and C leading to cirrhosis and portal hypertension are highly prevalent in Pakistan [11]. Many studies have reported the prevalence of EV as high as 50-60% among cirrhotic patients [12]. Studies on the predictive value of non-invasive indices regarding the presence of HRV remain scanty. Moreover, small-scale recent studies conducted have shown that platelet count, spleen diameter, and liver stiffness may also be used for stratifying the risk [13,14]. To date, no comprehensive study has compared the diagnostic performance of indices such as the P^2^MS index, EVendo score, international normalized ratio (INR) to platelet ratio, and splenic stiffness.

This gap underlines the need for a systematic evaluation of these indices, especially in populations with a high prevalence of chronic liver disease and scarce access to advanced health facilities. Therefore, the main aim of this study was to compare the diagnostic performance of the P^2^MS index, EVendo score, INR to platelet ratio (INPR), and splenic stiffness in predicting HRV in a Pakistani population.

## Materials and methods

Study design and setting

After the approval from the Ethical Review Committee, Sindh Institute of Urology and Transplantation (approval-1141), this cross-sectional study was carried out at the Department of Hepatogastroenterology from April 1, 2024, to September 30, 2024.

All the patients aged greater than 18 years and recently diagnosed with liver cirrhosis (as per operational definition (Table 1)) were enrolled in the study. While patients with a previous history of endoscopic intervention for EV, those with a history of hepatocellular cancer or other malignancies, those with a history of liver failure or thrombocytopenia or splenomegaly caused by hematological disorders, patients taking propranolol and other vasoactive medications, and those with portal vein thrombosis were excluded from the study.

**Table 1:** Operational definitions of cirrhosis of the liver, high-risk esophageal varices (HRVs), P2MS index, EVendo score, and INR to platelet ratio

Operational definitions
Cirrhosis of liver [14]: The patient with liver cirrhosis will be identified on abdominal ultrasound findings. The presence of three or more of the following findings will be considered as the presence of liver cirrhosis: 1. Altered echo texture of liver. 2. Irregular margins. 3. Spleen size more than 12 cm. 4. Portal vein diameter more than 12 mm. 5. Presence of free fluid in the abdomen on ultrasound.
High-risk esophageal varices (HRVs): Varices with a tendency to bleed [8]. Grade 1 - Small esophageal varices with red-wale sign. Grade 2 - Beading appearance of esophageal varices on endoscopy. Grade 3 - large, tortuous with a tumefactive appearance of varices on endoscopy, running in an oblique course
P^2^MS index [7]: The P^2^MS index was calculated using the formula: ((platelet count (×10^9^/L))^2^ / (monocyte fraction (%) × segmented neutrophil fraction (%))
EVendo score [8]: A = (8.5 × INR) + [AST (U/L) / 35) B = (platelet count (×10³/µL) / 150) + (BUN (mg/dL) / 20) + (hemoglobin (g/dL) / 15) EVendo score = (A / B) + 1 (if ascites present)
INR to platelet ratio (INPR) [15]: INPR = INR/platelet counts (×10^9^/L) × 100

Data collection procedure

After the informed consent, all the patients fulfilling the inclusion criteria were enrolled in the study. Each patient underwent laboratory investigations, including complete blood count and liver function tests followed by the ultrasound abdomen for features of chronic liver disease, splenomegaly, and splenic diameter (using TOSHIBA Aplio 50 Model MCM17545TS (Canon Medical Systems Corporation, Otawara, Japan)), and shear-wave elastography (SWE) to document the splenic stiffness. Esophagogastroduodenoscopy (EGD) was performed by a single gastroenterologist (with more than five years of experience in performing endoscopic interventions) in each patient to document the presence or absence of HRV.

Data analysis procedure

The data were entered and analyzed using SPSS (IBM SPSS Statistics for Windows, IBM Corp., Version 27.0, Armonk, NY). Continuous variables were expressed as mean ± SD, while categorical variables were expressed as frequencies and percentages. Continuous variables were analyzed using the Student t-test, while categorical variables were analyzed using the chi-square test. A p-value of ≤0.01 was considered statistically significant.

The area under the receiver operating curve (AUROC) was obtained for the P^2^MS index, EVendo score, INPR, and splenic stiffness in predicting HRV. At an optimal cutoff, the sensitivity, specificity, and diagnostic accuracy of each non-invasive score were obtained to predict HRV.

## Results

A total of 340 patients were included in the study. Out of them, 214 (62.9%) were males. The mean age was 44.9 ± 12.9 years. The most common cause of chronic liver disease was hepatitis C in 187 (55%) patients, followed by hepatitis B in 84 (24.7%) patients, autoimmune hepatitis in 34 (10%), alcoholic liver disease in 21 (6.2%) and hepatitis B and D co-infection in 11 (3.2%) patients. The cause of chronic liver disease was unknown in three (0.8%) patients. Out of 340 patients, Child-Turcotte-Pugh (CTP) class A and class B were observed in 231 (67.9%) and 10^9^ (32.1%) patients, respectively. Mean hemoglobin was 11.04 ± 2.1 g/dL, total leucocyte count (TLC) was 10.1 ± 4.1 × 10^9^/L, platelet was 93.2 ± 45.3 × 10^9^/L, INR was 1.2 ± 0.174, serum albumin was 3.5 ± 0.6 g/dL, aspartate transaminase (AST) was 55.8 ± 44.3, and alanine transaminase (ALT) was 78.1 ± 64.1. Mean splenic stiffness on SWE was 36.6 ± 20.6 kPa, EVendo score was 16.9 ± 5.6, INPR was 2.1 ± 2.6, and P^2^MS index was 17.8 ± 18.5. EV was observed in 184 (54.1%) patients, while HRV was noted in 84 (24.7%) patients (Table 2).

**Table 2:** Baseline characteristics of the studied population (n = 340) AIH - autoimmune hepatitis; ALT - alanine transaminase; AST - aspartate transaminase; CTP - Child-Turcotte-Pugh; HBV - hepatitis B; HCV - hepatitis C; HDV - hepatitis D; HRV - high-risk esophageal varices; INPR - INR-to-platelet ratio; INR - international normalized ratio; P^2^MS - ((platelet count (×10^9^/L))^2^ / (monocyte fraction (%) × segmented neutrophil fraction (%)); PLT - platelet; TLC - total leucocyte count

Variable		n (%)
Gender	Male	214 (62.9)
	Female	126 (37.1)
Cause of chronic liver disease	HCV	187 (55)
	HBV	84 (24.7)
	AIH	34 (10)
	HBV + HDV coinfection	11 (3.2)
	Alcohol	21 (6.2)
	Unknown	3 (0.8)
CTP class	A	231 (67.9)
	B	109 (32.1)
Esophageal varices	Yes	184 (54.1)
	No	156 (45.9)
HRV	Yes	84 (24.7)
	No	256 (75.3)
Age (years)		44.9 ± 12.9
Hemoglobin (g/dL)		11.04 ± 2.1
TLC (×10⁹/L)		10.1 ± 4.1
PLT (×10⁹/L)		177 ± 146
AST (IU/L)		55.8 ± 48.3
ALT (IU/L)		78.1 ± 64.1
Serum albumin (g/L)		3.5 ± 0.61
INR		1.2 ± 0.17
P^2^MS index		17.8 ± 18.5
EVendo score		16.9 ± 5.96
INPR		2.21 ± 2.6
Splenic stiffness (kPa)		36.6 ± 20.6

On comparative analysis, decreased hemoglobin (p ≤ 0.01), TLC (p ≤ 0.01), platelet count (p = 0.038), serum albumin (p = 0.012), P^2^MS index (p = 0.027) and increased AST (p ≤ 0.01), INR (p ≤ 0.01), splenic stiffness (p = 0.012), EVendo score (p ≤ 0.01) and INPR (p ≤ 0.01) were the factors significantly associated with the presence of HRV (Table 3).

**Table 3:** Comparison of baseline characteristics in predicting HRV (n = 340) ALT - alanine transaminase; AST - aspartate transaminase; CTP - Child-Turcotte-Pugh; HRV - high-risk esophageal varices; INPR - INR-to-platelet ratio; INR - international normalized ratio; P^2^MS - ((platelet count (×10^9^/L))^2^ / (monocyte fraction (%) × segmented neutrophil fraction (%)); PLT - platelet; TLC - total leucocyte count

Variable		High-risk esophageal varices		p-value
		Present (n = 84)	Absent (n = 256)	
Gender	Male	48 (57.1)	166 (64)	0.205
	Female	36 (42.9)	90 (36)	
CTP Class	A	47 (55.9)	184 (71.8)	0.007
	B	37 (44.1)	72 (28.2)	
Age (years)		34.4 ± 16.7	37.6 ± 16.4	0.214
Hemoglobin (g/dl)		10.2 ± 1.9	11.3 ± 2.1	≤ 0.001
TLC (×10⁹/L)		2.9 ± 1.0	6.1 ± 2.6	≤ 0.001
PLT (×10⁹/L)		51 ± 19	106 ± 42	0.038
AST (IU/L)		70 ± 38	41 ± 24	≤ 0.001
ALT (IU/L)		57 ± 30	38 ± 30	0.214
Serum albumin (g/L)		3.2 ± 0.4	3.5 ± 0.6	0.012
INR		1.3 ± 0.13	1.1 ± 0.16	≤ 0.001
Splenic stiffness (kPa)		54.7 ± 27.5	30.3 ± 13	0.012
EVendo score		21.3 ± 5.6	15.5 ± 5.3	≤ 0.001
INPR		3.0 ± 1.98	1.9 ± 2.7	≤ 0.001
P^2^MS index		4.3 ± 4.4	22.1 ± 19.1	0.027

**Figure 1:**
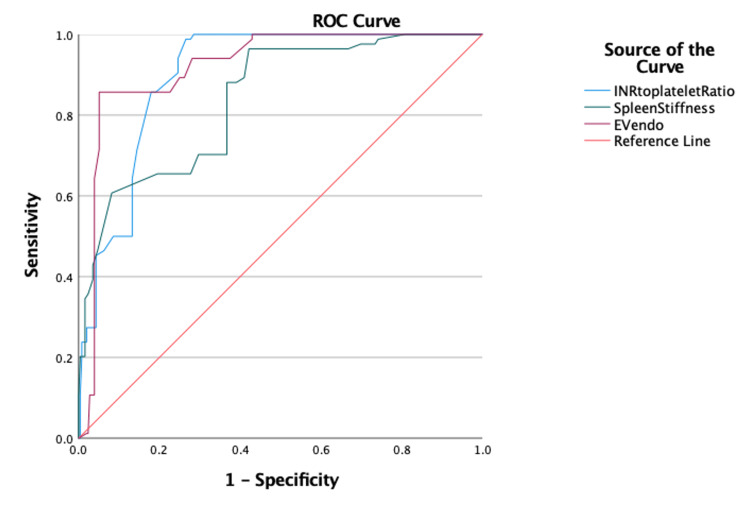
The area under the receiver operating curve (AUROC) for INPR, splenic stiffness, and EVendo score in predicting HRV was 0.90 (p ≤ 0.001), 0.838 (p ≤ 0.001), and 0.92 (p ≤ 0.001), respectively. HRV - high-risk esophageal varices; INPR - INR to platelet ratio

**Figure 2:**
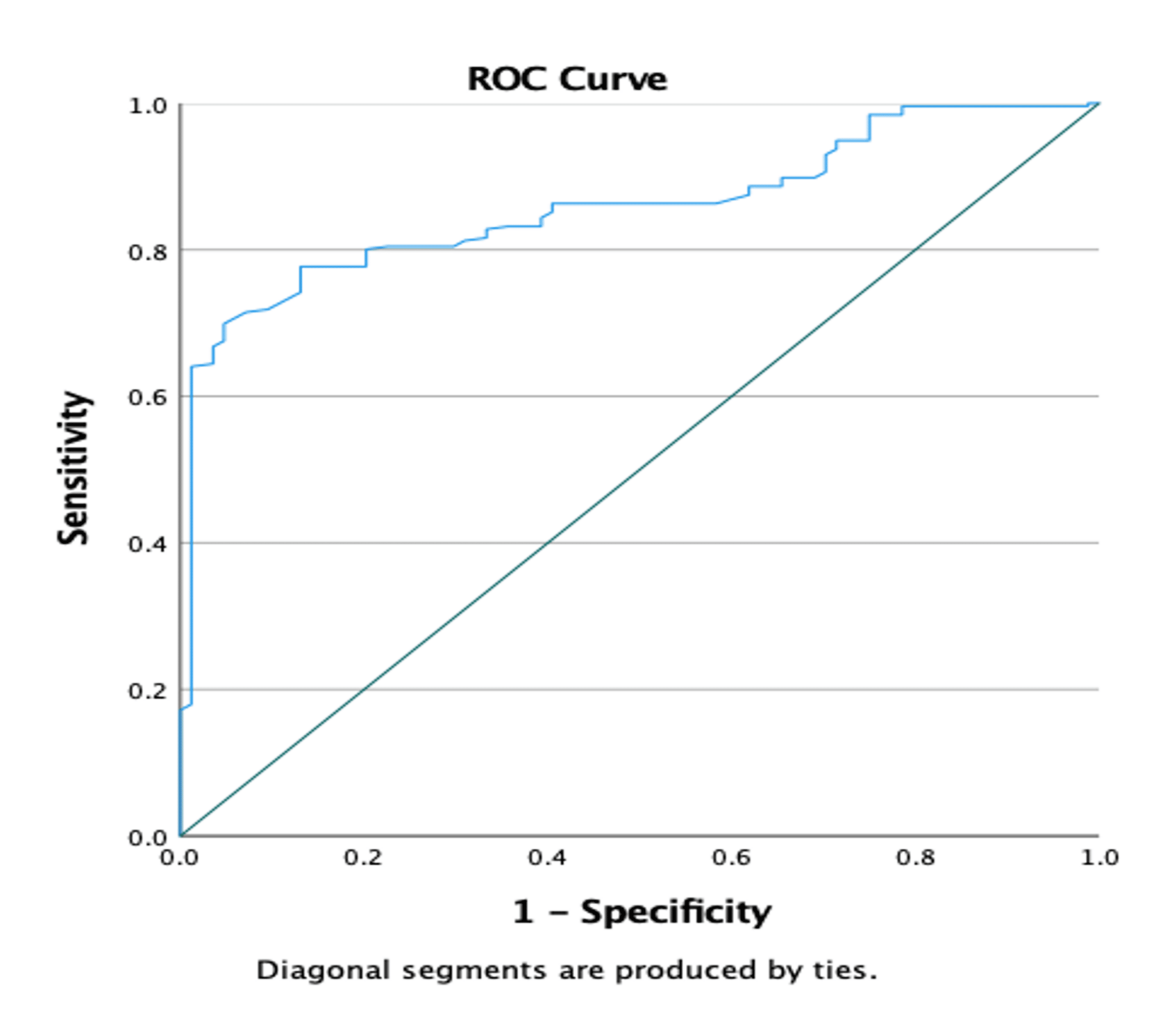
The area under the receiver operating curve (AUROC) for the P2MS index in predicting HRV was 0.859 (p ≤ 0.01). HRV- high-risk esophageal varices; P^2^MS - ((platelet count (×10^9^/L))^2^ / (monocyte fraction (%) × segmented neutrophil fraction (%))

AUROC was highest for the EVendo score (AUROC = 0.92, p ≤ 0.001), followed by INPR (AUROC = 0.90, p ≤ 0.001), the P^2^MS index (AUROC = 0.859, p ≤ 0.01), and splenic stiffness (AUROC = 0.838, p ≤ 0.001) in predicting HRV in patients with chronic liver disease (Figure 1 and Figure 2). The diagnostic accuracy was highest for the EVendo score (86.56%) in predicting HRV, followed by the P^2^MS ratio (81.71%), splenic stiffness (66.47%), and INPR (58.24%) (Table 4).

**Table 4:** Diagnostic accuracy of different scores in predicting HRV AUROC - area under the receiver operating curve; HRV - high-risk esophageal varices; INPR - INR-to-platelet ratio; NPV - negative predictive value; P^2^MS - ((platelet count (×10^9^/L))^2^ / (monocyte fraction (%) × segmented neutrophil fraction (%)); PPV - positive predictive value

Predictive model	AUROC	p-value	Cutoff	Sensitivity	Specificity	PPV	NPV	Diagnostic accuracy
EVendo score	0.92	≤ 0.01	≥15.7	92.94 %	71.88%	52.34%	96.84%	86.56 %
INPR	0.90	≤ 0.01	≥1.5	92.86 %	58.98 %	36.95%	95.24 %	58.24 %
P^2^MS index	0.859	≤ 0.01	≤ 5.4	77.38 %	80.47 %	56.52 %	91.56 %	81.71 %
Spleen stiffness	0.838	≤ 0.01	≥ 33	89.29 %	46.88 %	41.67%	66.47 %	66.47 %

## Discussion

This study aimed to evaluate and compare the diagnostic performance of various non-invasive indices - EVendo score, INPR, P^2^MS index, and splenic stiffness - in predicting HRV in a Pakistani population with chronic liver disease. The findings provide critical insights into the utility and limitations of these indices, particularly in resource-constrained settings where invasive diagnostic methods, such as endoscopy, may not be feasible for widespread use.

The findings of this study are consistent with prior research that has demonstrated the potential of non-invasive methods in predicting HRV. Several earlier studies have evaluated the role of platelet count, spleen size, and liver stiffness as predictors of EV [13,14]. However, this study goes a step further by systematically comparing the diagnostic performance of four indices in a single population, providing a comprehensive assessment of their relative efficacy.

Previously, Jan et al. reported an AUROC of 0.852 for the EVendo score in predicting HRV with excellent diagnostic accuracy of 84.19%, highlighting its reliability in clinical practice [8]. In our study, the AUROC of the EVendo score was slightly higher at 0.92, with a higher diagnostic accuracy of 86.56% in predicting HRV, reflecting the population-specific factors, such as the predominance of viral hepatitis as an etiology of chronic liver disease in a Pakistani cohort [11]. While the EVendo score has not been as extensively validated in international literature, this finding underscores its potential as a universal predictor of HRV. Its incorporation of clinical and biochemical parameters, such as INR, platelet count, and AST, provides a comprehensive assessment of risk [8].

In the previous literature, INPR has never been used to predict HRV [15]. Previously, Rizvi et al. reported an AUROC of 0.823 of platelet to prothrombin time ratio in predicting EV with an excellent sensitivity of 97.8% and a good specificity of 83% [16]. However, the diagnostic accuracy of this ratio was not mentioned in the index study. We evaluated the INPR ratio for the prediction of HRV, and it showed an AUROC of 0.90 with an excellent sensitivity in predicting HRV. However, it lacked specificity and diagnostic accuracy in predicting HRV.

In our study, the P^2^MS index demonstrated an AUROC of 0.859 with good specificity and diagnostic accuracy in predicting HRV. In comparison, a study by Ali et al. in a Pakistani population reported an AUROC of 0.84 with good sensitivity and specificity of more than 80%, respectively, and slightly lower diagnostic accuracy of 80% as compared to our study, which showed a diagnostic accuracy of 81.71% [7]. The slight variations could be attributed to the differences in sample size, population demographics, and underlying etiologies of liver disease, as in the former, only the patients with HCV-related chronic liver disease were enrolled.

Spleen stiffness measured by SWE has been widely investigated as a surrogate marker for portal hypertension. Previously, Takuma et al. demonstrated an AUROC of 0.955 for splenic stiffness in predicting HRV with excellent accuracy in ruling out portal hypertension, EV, and HRV [17]. Similarly, Hu et al. demonstrated the role of spleen stiffness in predicting portal hypertension and EV [9]. However, a meta-analysis done by Jachs and his colleagues in evaluating the role of spleen stiffness in estimating portal hypertension and EV reported a pooled specificity of 66% in predicting HRV [18]. Here, we report an AUROC of 0.838 for spleen stiffness, corroborating its utility in non-invasive diagnostics. However, it lacked specificity (46.88%) and diagnostic accuracy (66.47%) compared to the other indices, suggesting that its role may be more complementary than primary in predicting HRV.

There are several limitations to this study. At first, the cross-sectional design precludes the assessment of longitudinal outcomes, such as the progression of HRV or the impact of early detection on clinical outcomes. Secondly, this single-centered study type may limit the generalizability of the findings to other regions or healthcare settings. This study’s strengths include its robust methodological design, large sample size, and comprehensive comparison of multiple indices. By focusing on a Pakistani population, the study addresses a critical gap in the literature, as most prior research has been conducted in Western and East Asian settings. The inclusion of diverse etiologies of chronic liver disease, predominantly hepatitis B and C, enhances the generalizability of the findings to similar populations in resource-limited settings.

## Conclusions

The results from this study have shown promise regarding the applicability of the non-invasive indices in predicting EV in a Pakistani population with chronic liver disease. The EVendo score showed the highest diagnostic accuracy among all the indices analyzed for the diagnosis of HRV and may, therefore, be considered a strong tool for clinical use, even in resource-limited settings. These findings indicate that population-specific approaches are crucial in the assessment of diagnostic tools, particularly in resource-limited populations like Pakistan. Further longitudinal assessment in a multi-center setting will be required not only to validate the role of these non-invasive indices in predicting HRV but also to assess their general applicability to broad clinical use in resource-poor health settings.
